# Psychologists’ knowledge, attitudes, and training experiences regarding mental illness in individuals with intellectual disabilities in Saudi Arabia: a cross-sectional study

**DOI:** 10.3389/fpsyt.2026.1764215

**Published:** 2026-10-06

**Authors:** Abeer AL Lihabi

**Affiliations:** Department of Psychiatry, Taibah University, Madinah Almunwarah, Saudi Arabia

**Keywords:** attitudes, intellectual disabilities, mental illness, perceptions, services

## Abstract

**Introduction:**

Intellectual disability (ID) impacts an estimated 1-3% of the population worldwide, with a higher incidence in regions with limited healthcare access. In Saudi Arabia, current prevalence data, especially for adults, are scarce. This cross-sectional study explores the systemic challenges psychologists perceived in the diagnosis and treatment of mental illnesses in individuals with intellectual disabilities.

**Methods:**

A standardized Arabic-language questionnaire was distributed to 144 licensed psychologists in Saudi Arabia to collect data on professional backgrounds, educational history, work experience, attitudes, and knowledge.

**Results:**

Data were analyzed using factorial ANOVA and Spearman’s correlation tests. The analysis revealed that years of professional experience significantly influenced attitudes (F = 5.024, p =.008, partial η² =.073), while training experience had a pronounced effect on knowledge of mental comorbidities in ID (F = 75.83, p <.001, partial η² =.367). A strong correlation was identified between training experience and knowledge (ρ =.811, p <.001), whereas no significant correlation was found for attitudes.

## 1 Introduction

Globally, up to 3% of the population lives with intellectual impairment of varying severity. Intellectual disability refers to a disorder that starts before the age of 18 and is marked by notable limits in both intellectual performance and adaptive behavior. Prevalence is particularly high in economically disadvantaged regions primarily due to restricted access to healthcare services, such as early diagnostic and intervention services (1). Regarding international trends, a previous study reported a prevalence rate of 8.9 per 1,000 children with intellectual disability (ID) in Saudi Arabia (2). Nevertheless, there is a lack of research evaluating the prevalence of ID across all age groups, particularly among adults. Recent studies have targeted specific subgroups or broader disability categories. According to data from the Saudi General Authority for Statistics, 2.9% of the population reported experiencing severe impairment, although this did not specifically address intellectual disability (3). Other studies have provided broader disability prevalence statistics and investigated general risk factors and aspects of ID in preschool-aged children (4–6). Despite these efforts, a substantial research gap persists concerning the overall prevalence of ID, especially among adults in Saudi Arabia, emphasizing the need for further research (7, 8).

Individuals with intellectual disabilities are disproportionately affected by mental illness, with prevalence estimates ranging from 33% to 75% (9–11). This is in sharp contrast to the general population, where global data indicate that 12 to 17% of individuals experience mental health issues annually (12, 13). In the United States, approximately 23% of adults are affected by mental disorders (12, 14, 15). This pronounced disparity highlights the critical need for tailored mental health services and research for this vulnerable group, as individuals with intellectual disabilities are at least two to three times more likely to face mental health challenges than their neurotypical peers.

Effective policy planning and the development of training interventions require an understanding of barriers, such as educational background, familiarity with intellectual disabilities, workplace conditions, and regional influences, including prevailing attitudes, beliefs, and practices. The World Health Organization’s remarks are released concurrently with Saudi Arabia’s mental health policies, which have a clear deterrent stance and are prepared in terms of efficiency (Carlisle, 2018) (27) highlighting the need for more research, especially on the obstacles psychologists encounter when attempting to diagnose and treat mental diseases in this Saudi Arabian population.

The study examines how variables such as training level, years of experience, familiarity with individuals with intellectual disabilities, and regional differences affect psychologists’ attitudes and perceptions of systemic deficiencies. The key research questions included the following:

1. What barriers do professionals identify during the diagnosis and treatment of individuals with intellectual disabilities?
2. Do attitudes and beliefs differ according to training level, job type, and experience?
3. Are there regional variations in the perception of systemic neglect?
4. Is diagnostic overshadowing a commonly held belief among professionals?

Therefore, this study mainly aimed to address these challenges by investigating the perspectives of Saudi Arabian psychologists on the to diagnosing and treating mental illness in individuals with intellectual disabilities.

The study’s premise is that psychologists with more specialized training and clinical experience will have better understanding and more favorable views about diagnosing and treating mental diseases in people with intellectual disability.

## 2 Methods

### 2.1 Study design

This cross-sectional study utilized a structured self-report questionnaire to collect objective background data, such as demographics, work environment, and training, as well as subjective insights, including attitudes, beliefs, and perceived challenges.

### 2.2 Participant characteristics

The study participants included licensed clinical psychologists, counseling psychologists, and academic psychologists. To be eligible for inclusion, participants were required to i) obtain at least a bachelor’s degree in psychology; ii) be currently employed in clinical, counseling, or academic roles; and iii) consent to participate in the study. Participation was voluntary and anonymous. Participants were informed that by completing the survey, they provided consent for their responses to be utilized for research purposes.

### 2.3 Study sample size calculation

*A priori* power analysis was performed to determine the appropriate sample size for this study, which involved a one-way ANOVA with five distinct groups, using G*Power (version 3.1.9.7). The parameters for this analysis included a significance level of α=0.05, a projected medium effect size (f = 0.3), and a target statistical power of 0.824. This analysis determined that a sample size of 144 participants was required, ensuring an 82.4% chance of detecting statistically significant differences among the groups, assuming the expected effect size is present. Therefore, the sample size was sufficient to provide reliable statistical outcomes for this study.

### 2.4 Measures

A structured questionnaire in Arabic was designed following a comprehensive review of the relevant literature and expert consultations with all psychiatrists, one of whom specialized in dual diagnosis. Three specialists assessed the questionnaire, which was refined based on their feedback. Initially, it was piloted with a sample of ten participants, and its validity was assessed using Cronbach’s alpha. After removing one item, the alpha value improved to 0.7, confirming its validity, and the questionnaire was subsequently distributed.

The instrument was structured into several sections:

- Demographics: This section collected details of participant age, gender, years of experience, and work region which was categorized into the main regions of Saudi Arabia.
- Training and professional exposure: This section explored the type of training participants had received, the nature of their current role (clinical, academic, or a combination of both), and the degree of exposure to individuals with intellectual disabilities during education, clinical training, and current practice.
- Attitudes and knowledge: This section featured a series of Likert-scale statements (1 = Strongly Disagree, 5 = Strongly Agree) to assess beliefs regarding psychiatric diagnosis, treatment effectiveness, diagnostic overshadowing, and perceived systemic neglect.
- Knowledge assessment: Participants were tasked to answer multiple-response questions to identify psychiatric disorders that are frequently associated with intellectual disabilities.

### 2.5 Details

#### 2.5.1 Scale structure at

(**Table 1**)

**Table 1:** Structure and response format of the questionnaire dimensions.

Dimension	Item numbers	No. of items	Response format
Training	Items 1–5	5	None/Weak/Medium/High (4-point)
Knowledge	Items 6–12	7	Very Easy to Difficult (4-pt) + 5-pt Likert (Item 12)
Attitudes	Items 13–22	10	Strongly Disagree to Agree (5-point Likert) Strongly

#### 2.5.2 Dimension 1: training (items 1–5)

This dimension assesses the degree to which respondents have encountered people with intellectual disabilities at various points in their academic and professional lives, as well as the challenges they face in the workplace. A four-point rating system is used to record responses: None (1), Weak (2), Medium (3), and High (4).

Score Interpretation: A high score on Items 1–3 indicates greater exposure to people with intellectual disabilities and is a favorable sign of professional experience. Greater perceived difficulty in interactions is indicated by high scores on Items 4–5, which suggest that additional training and support are needed (**Table 2**).

**Table 2:** Training dimension (Items 1–5).

Item no.	Question
1	Exposure to individuals with intellectual disabilities during the academic study stage
2	Exposure to individuals with intellectual disabilities during the practical training stage
3	Exposure to individuals with intellectual disabilities in the current workplace
4	Difficulty in dealing with the families of individuals with intellectual disabilities
5	Difficulty in meeting or interacting with individuals with intellectual disabilities

#### 2.5.3 Dimension 2: knowledge (items 6–12)

The respondents’ theoretical and clinical knowledge of intellectual disability, including diagnosis, symptomatology, and the recognition of co-occurring mental illnesses, is assessed in this domain. A 4-point perceived difficulty scale is used to score items 6 through 11: Very Easy (4), Easy (3), Medium Difficulty (2), and Difficult (1). A typical 5-point Likert agreement scale is used for item 12: Strongly Disagree (1) to Strongly Agree (5).

Score interpretation: A high score for Items 6–11 denotes easier application of knowledge, which is a good sign. A high score (agreement) on Item 12 indicates a clinical misperception and is therefore a negative indication because it implies the professional is unable to differentiate between concomitant psychiatric illnesses and intellectual disability (**Table 3**).

**Table 3:** Knowledge dimension (Items 6–12).

Item no.	Question
6	I find it [easy/difficult] to recognize the basics of diagnosing intellectual disabilities
7	I find it [easy/difficult] to know the tests used in diagnosing intellectual disabilities
8	I find it [easy/difficult] to know the symptoms and traits of intellectual disabilities
9	I find it [easy/difficult] to know the symptoms of psychiatric disorders in individuals with intellectual disabilities
10	I find it [easy/difficult] to identify the potential presence of a comorbid psychiatric disorder alongside an intellectual disability
11	I consider making a referral decision when a psychiatric illness is suspected to be [easy/difficult]
12	All psychological changes that occur in individuals with intellectual disabilities are attributed to the intellectual disability itself

#### 2.5.4 Dimension 3: attitudes (items 13–22)

This dimension investigates the therapeutic viewpoints, assumptions, and attitudes of professionals regarding mental health concerns in people with intellectual impairments. A 5-point Likert scale is used for each item: Strongly Disagree (1), Disagree (2), Neutral (3), Agree (4), and Strongly Agree (5).

Score interpretation: Within this dimension, a high score is not consistently positive or negative — it depends on the item’s direction. Since items 14, 15, 16, 17, and 18 include positive wording, a high score indicates accurate and knowledgeable attitudes. When calculating a total Attitudes score, items 13, 19, 20, 21, and 22 should be reverse scored because they are negatively written. This way, a higher overall score consistently indicates more correct and favorable professional attitudes (**Table 4**).

**Table 4:** Attitudes dimension (Items 13–22).

Item no.	Question	High score meaning
13	Intellectual disability is the same as mental illness	Negative (misconception) — reverse scored
14	Individuals with intellectual disabilities are susceptible to mental illness	Positive (accurate belief)
15	Individuals with intellectual disabilities are more prone to mental illness	Positive (accurate belief)
16	There is a difference between mental illnesses and behavioral changes	Positive (accurate belief)
17	Medications can help treat mental illnesses in individuals with intellectual disabilities	Positive (accurate belief)
18	Behavioral therapy can be effective in treating psychological disorders in individuals with intellectual disabilities	Positive (accurate belief)
19	It is difficult to identify and distinguish mental illnesses in individuals with intellectual disabilities	Negative (reflects a limitation) — reverse scored
20	There is a tendency to neglect the diagnosis of psychological disorders in individuals with intellectual disabilities	Negative (reflects systemic neglect) — reverse scored
21	There is a tendency to neglect the treatment of psychological disorders in individuals with intellectual disabilities	Negative (reflects systemic neglect) — reverse scored
22	The emergence of difficult behavioral changes is a characteristic symptom of intellectual disability	Negative (misconception) — reverse scored

#### 2.5.5 Based on their overall subscale score

Participants were divided into low and high groups for each dimension using a median split. The median was 13 for the Training dimension (possible range: 5–20), 18 for the Knowledge dimension (possible range: 6–29), and 34 for the Attitudes dimension (possible range: 10–50). The low group consisted of participants who scored at or below the median, and the high group consisted of those who scored above the median.

### 2.6 Statistical analysis

The analysis of the data was performed using SPSS version 26.0. Categorical variables were reported as frequencies and percentages. Differences in knowledge and attitudes concerning psychiatric disorders among individuals with intellectual disabilities were evaluated using factorial ANOVA, while correlations between variables were assessed using Spearman’s rho. Statistical significance was defined as a p-value of less than 0.05, with a confidence interval set at 95% (95%CI). 95% confidence intervals were computed for all group means using the t-distribution formula: CI = M ± t(0.975, df = n − 1) × (SD/√n). For significant ANOVA results, Bonferroni-corrected pairwise comparisons were conducted to identify specific group differences while controlling for Type I error across multiple comparisons.

## 3 Results

The sample consisted of 144 participants of clinical psychologists in Saudi Arabia. **Table 5** illustrates the demographic and professional characteristics of the sample, The sample comprised mostly females (71.5%), with the 31–40 age group most represented (40.3%). The majority held a Bachelor’s or Master’s degree (approximately 84%), and work experience varied, with 41% possessing over 10 years of experience. Most participants worked in the Central and Western Regions (around 85%) with both children and adults (47.9). **Tables 6**–**9**: Participants’ attitudes differed significantly by experience level, *F* = 6.989, *p* = .001. Participants with more than 10 years of experience had significantly lower attitude scores than those with less than 5 years (*p* = .001) and 5–10 years (*p* = .008). No significant difference was found between the latter two groups.

**Table 5:** Demographic and professional characteristics of the participants (N = 144).

Variable	Subgroup	n (%)	Knowledge M (SD)	Knowledge 95% CI	Knowledge F (p)	Attitude M (SD)	Attitude 95% CI	Attitude F (p)
Gender	Male	41 (28.5%)	13.02 (2.96)	[12.09, 13.95]	.100 (.752)	37.41 (3.02)	[36.46, 38.36]	.324 (.570)
	Female	103 (71.5%)	13.17 (2.40)	[12.70, 13.64]		37.09 (3.15)	[36.47, 37.71]	
Age	20–30	44 (30.6%)	13.09 (2.69)	[12.27, 13.91]	1.094 (.354)	38.02 (3.39)	[36.99, 39.05]	2.587 (.056)
	31–40	58 (40.3%)	12.93 (2.15)	[12.36, 13.50]		37.22 (2.74)	[36.50, 37.94]	
	41–50	36 (25.0%)	13.69 (2.63)	[12.80, 14.58]		36.36 (3.30)	[35.24, 37.48]	
	Above 50	6 (4.2%)	12.00 (4.47)	[7.31, 16.69]		35.50 (1.23)	[34.21, 36.79]	
Academic Degree	Institute	1 (0.7%)	17.00 (—)	n=1, not estimable		37.00 (—)	n=1, not estimable	
	Bachelor’s	62 (43.1%)	13.45 (2.51)	[12.81, 14.09]	1.488 (.220)	36.98 (3.39)	[36.12, 37.84]	.207 (.891)
	Master’s	59 (41.0%)	12.78 (2.44)	[12.14, 13.42]		37.42 (3.05)	[36.63, 38.21]	
	Doctorate/PhD	22 (15.3%)	13.00 (2.91)	[11.71, 14.29]		37.09 (2.52)	[35.97, 38.21]	
Years of Exp.	≤5 years	54 (37.5%)	13.24 (2.78)	[12.48, 14.00]	.928 (.398)	38.02 (3.27)	[37.13, 38.91]	6.989 (.001)*
	5–10 years	31 (21.5%)	12.58 (2.60)	[11.63, 13.53]		37.84 (2.52)	[36.92, 38.76]	
	>10 years	59 (41.0%)	13.32 (2.33)	[12.71, 13.93]		36.07 (2.93)	[35.31, 36.83]	
Region	Southern	12 (8.3%)	12.50 (3.23)	[10.45, 14.55]	.225 (.924)	38.00 (3.39)	[35.85, 40.15]	2.468 (.048)*
	Northern	5 (3.5%)	13.20 (2.68)	[9.87, 16.53]		38.00 (3.94)	[33.11, 42.89]	
	Western	55 (38.2%)	13.22 (2.77)	[12.47, 13.97]		36.65 (2.92)	[35.86, 37.44]	
	Central†	67 (46.5%)	13.18 (2.28)	[12.62, 13.74]		37.64 (3.02)	[36.90, 38.38]	
	Eastern	5 (3.5%)	12.80 (2.17)	[10.11, 15.49]		34.00 (3.08)	[30.18, 37.82]	
Patient Category	Children	17 (11.8%)	13.88 (2.18)	[12.76, 15.00]	1.441 (.240)	37.06 (2.93)	[35.55, 38.57]	.015 (.985)
	Adults	58 (40.3%)	12.76 (2.92)	[11.99, 13.53]		37.21 (3.12)	[36.39, 38.03]	
	Children & Adults	69 (47.9%)	13.26 (2.30)	[12.71, 13.81]		37.19 (3.18)	[36.43, 37.95]	

**Table 6:** T-test of knowledge regarding psychiatric disorders in individuals with intellectual disabilities by training experience.

Source	Group	N	Mean (± SD)	T	P-value
Training experience	Low	69	11.5 (2.2)	9.62	<.001
	high	75	14.7 (1.7)		

P-value is statistically significant.

An independent samples t-test revealed that participants with high training experience (M = 14.7, SD = 1.7) scored significantly higher on knowledge of psychiatric disorders in individuals with intellectual disabilities than those with low training experience (M = 11.5, SD = 2.2), t (142) = 9.62, p <.001.

**Table 7:** Two-way ANOVA of attitudes toward psychiatric disorders among individuals with intellectual disabilities.

Source	Group	Mean (± SD)	Sum of squares (SS)	df	Mean square (MS)	F	P-value	Partial η²
Knowledge	Low	36.9 (3.0)	10.49	1	10.49	1.08	.300	.008
	high	37.5 (3.27)						
Training experience	Low	37.2 (3.1)	23.62	1	23.62	2.44	.120	.017
	high	37.2 (3.1)						
Knowledge* Training experience			5.04	1	5.04	.521	.472	.004
Error	1353.915	140	9.671					
Total	200444.00	144						

P-value is statistically significant.

Two-way ANOVA showed no significant main effects of knowledge or training experience on attitudes toward psychiatric disorders, and their interaction was also non-significant.

**Table 8:** *Post hoc* comparisons — attitudes by years of experience (Bonferroni-Corrected).

Comparison	Mean diff	SE	T	P (uncorrected)	P (Bonferroni)	Significant?
≤5 years vs. 5–10 years	0.18	0.672	0.27	.789	1.000	No
≤5 years vs. >10 years	1.95	0.562	3.47	.001	.002	Yes
5–10 years vs. >10 years	1.77	0.662	2.67	.008	.025	Yes

Psychologists with more than 10 years of experience had significantly lower attitude scores than those with less than 5 years of experience (p = .002) and those with 5–10 years of experience (p = .025). No significant difference was found between the ≤5 years and 5–10 years groups (p = 1.000).

**Table 9:** *Post hoc* comparisons — attitudes by region of work (Bonferroni-Corrected).

Comparison	Mean diff	SE	T	P (uncorrected)	P (Bonferroni)	Significant?
Southern vs. Northern	0.00	1.621	0.00	1.000	1.000	No
Southern vs. Western	1.35	0.970	1.39	.166	1.000	No
Southern vs. Central	0.36	0.954	0.38	.707	1.000	No
Southern vs. Eastern	4.00	1.621	2.47	.015	.148	No
Northern vs. Western	1.35	1.422	0.95	.344	1.000	No
Northern vs. Central	0.36	1.412	0.26	.799	1.000	No
Northern vs. Eastern	4.00	1.926	2.08	.040	.396	No
Western vs. Central	−0.99	0.554	−1.79	.076	.761	No
Western vs. Eastern	2.65	1.422	1.86	.065	.646	No
Central vs. Eastern	3.64	1.412	2.58	.011	.110	No

While the omnibus ANOVA was statistically significant, no individual pairwise comparison survived Bonferroni correction. The Eastern region consistently showed the lowest attitude scores (M = 34.00), and the largest uncorrected differences were observed between the Eastern region and the Northern (Δ = 4.00, p = .040), Southern (Δ = 4.00, p = .015), and Central (Δ = 3.64, p = .011) regions — though none survived correction for multiple comparisons.

Participants’ attitudes differed significantly by region of work, F = 2.658, p = .048. Participants working in the northern (p = .04), southern (p = .02) and central (p = .01) regions had significantly higher attitude scores than those working in the eastern region. No other regional differences were statistically significant.

Spearman’s correlation analysis revealed a strong positive association between knowledge of psychiatric disorders (including diagnosis, treatment, diagnostic overshadowing, and perceived systemic neglect) in individuals with intellectual disabilities and clinical training experience (ρ = .811, p <.001). In contrast, attitude showed no significant correlation with either knowledge (ρ = .093, p = .266) or training experience (ρ = .041, p = .626).

## 4 Discussion

This cross-sectional study explored Saudi Arabian psychologists’ perspectives on systemic barriers to the diagnosis and treatment of mental disorders in individuals with intellectual disabilities. These findings offer insights into how training, professional experience, and contextual factors influence knowledge, attitudes, and therapeutic practices in this population. These results highlight the importance of training experience in enhancing psychologists’ understanding of mental disorders that co-occur with intellectual disabilities. The two-way ANOVA revealed a statistically significant and large effect size (partial η² = 0.367), indicating that psychologists with more professional training were better equipped to identify and understand the mental health conditions of individuals with intellectual disabilities.

These findings are in line with other studies indicating that exposure and professional training greatly improve the diagnostic skills of doctors and reduce reliance on diagnostic stereotypes or overshadowing (16–18). The absence of notable differences across degrees, professions, and regions suggests that clinical training is a key factor in developing diagnostic competency rather than merely academic credentials. Professional experience had a significant impact on attitudes toward individuals with ID; psychologists with over a decade of experience demonstrated considerably less positive attitudes than their less experienced peers.This may be related to earlier educational curricula that provided limited training on mental health in individuals with intellectual disabilities, while prolonged exposure to systemic barriers and limited resources may foster a more cautious clinical outlook.

This is consistent with previous research indicating that prolonged exposure to complex cases can foster empathy and confidence, thereby enhancing engagement and diminishing stigmatization of clients with intellectual disabilities (19). These findings may suggest a gradual shift in attitudes as psychologists become more familiar with the unique challenges associated with this population. However, the absence of a direct correlation between knowledge and attitudes suggests that simply acquiring knowledge may not be sufficient to alter attitudes, highlighting the importance of incorporating experiential and psychosocial learning strategies into professional development programs (20). The disconnect between training and attitudes may be explained by the deeply rooted nature of professional biases and cultural beliefs, which are not easily modified by didactic training alone. Attitude change requires sustained, reflective, and experiential learning approaches. Diagnostic overshadowing, identified as a persistent challenge in the literature, is another significant finding (21, 22).

It is important to acknowledge that this study did not directly assess misdiagnosis rates, although the observed inconsistencies in attitudes and variability in knowledge scores indicate that diagnostic overshadowing likely persists as a barrier to clinical practice. Therefore, conclusions regarding the prevalence of diagnostic overshadowing based solely on these data should be approached with caution. The lack of interdisciplinary teamwork and limited exposure to patients with dual-diagnoses during training — issues frequently cited in similar contexts — may contribute to this phenomenon (23). The strong correlation between training experience and knowledge further supports the notion that psychologists with structured, specialized training are more adept at differentiating between mental illness and intellectual impairment, thus reducing the impact of diagnostic overshadowing.

The clustering of responders in the Central and Western regions highlights a geographical disparity in workforce allocation, which when considered systemically, represents broader healthcare inequities in Saudi Arabia. The shortage of psychologists in remote areas, such as the Northern and Eastern regions, could indicate that access to psychological services and opportunities for specialized training are limited in these regions. These findings are consistent with international research indicating that individuals with ID have higher rates of undiagnosed mental health issues in less developed areas, partly due to insufficient professional support systems (11, 16, 19).

The data presented in **Figure 1** illustrate the proportion of psychologists who participated in the survey and reported that mental disorders were prevalent in their professional practice. The findings indicate a broad consensus among professionals that depression is the most common disorder (69%), followed by ADHD (56%) and schizophrenia (nearly 50%). This perspective aligns with international research, which consistently identifies depression as one of the most prevalent mental health issues worldwide, and is often linked to a range of biological and psychosocial factors. The heightened awareness and improved diagnostic detection of attention-related disorders in both adults and children may account for the significant ranking of ADHD in the current data. Social and generalized anxiety disorders were also perceived as prevalent (49% and 48%, respectively), remaining consistent with other studies indicating that anxiety-related disorders are among the most frequently reported psychiatric issues. The episodic character of bipolar disorder and the requirement for specialized evaluation for accurate diagnosis may contribute to its relatively low perception (36%). These disorders are often viewed as comorbidities or manifestations of underlying mental disorders rather than as distinct illnesses. Similarly, schizophrenia, although approximately 50% of respondents reported encountering the disorder, remains less common than mood and anxiety disorders, possibly due to its lower prevalence in the general population. Substance abuse and suicide were considered relatively common (28% and 32%, respectively). This may suggest that, despite their significance, these issues are frequently perceived as comorbidities or consequences of underlying mental illnesses rather than independent disorders. Overall, these findings suggest that mood and attention-related disorders are the most prevalent mental health issues encountered and identified by psychologists in this study.

**Figure 1:**
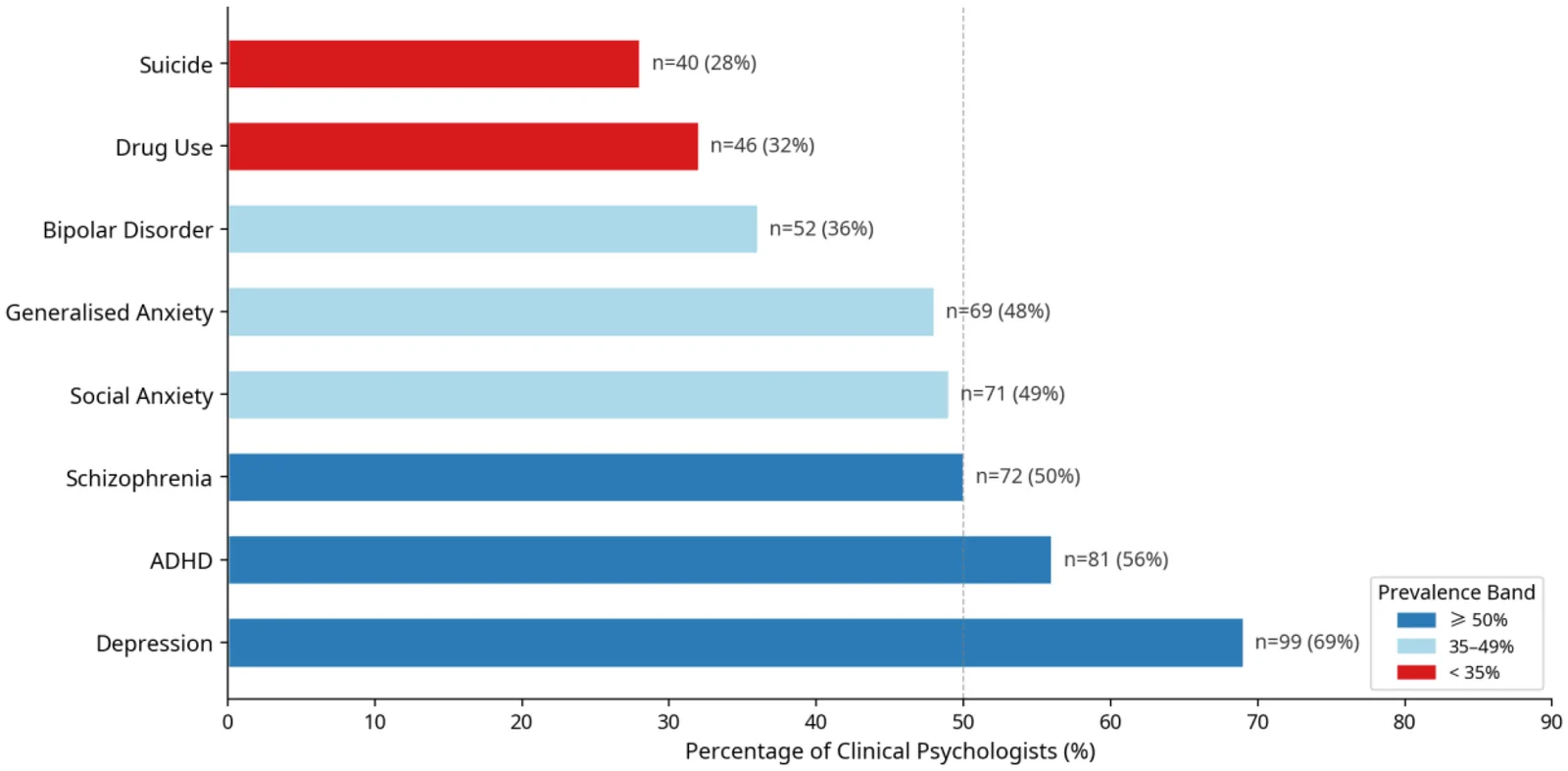
Most common psychiatric disorders with intellectual disabilities according to clinical psychologists (N = 144). Their feedback indicated that depression was perceived as the most widespread disorder (69%), followed by attention deficit hyperactivity disorder (56%) and schizophrenia (50%). Social anxiety and generalized anxiety were also commonly cited (49% and 48%, respectively). Suicide and drug use were considered to have moderate prevalence (28% and 32%, respectively). In contrast, bipolar disorder was perceived to be less prevalent (36%). These results suggest that psychologists generally perceive mood- and attention-related disorders as the most common psychological issues, whereas more severe conditions, such as bipolar disorder and schizophrenia, are perceived as less frequent.

A statistically flawed comparison emerges when clinical judgments are directly juxtaposed with contemporary epidemiological studies of the actual prevalence of these disorders within the ID community. As a result, the observed discrepancy serves as a critical lens for examining the methodological biases inherent in clinical practice rather than being perceived merely as a statistical comparison.

The inadequate detection of substance use and bipolar disorder, which may be related to actual prevalence variations or diagnostic caution among doctors, highlights the need for enhanced screening tools and culturally sensitive diagnostic guidelines. This study identifies systemic challenges, such as insufficient interdisciplinary collaboration, uneven geographic distribution of expertise, and a lack of specialized training — as persistent barriers to the accurate diagnosis and effective treatment of mental health conditions in individuals with intellectual disabilities. A comprehensive strategy is necessary to address these issues, including the integration of modules specific to intellectual disabilities in both undergraduate and graduate psychology programs. Additionally, ongoing professional development programs offering practical training for dual diagnosis should be established. Strengthening collaboration between mental health and disability care providers is essential. Moreover, it is important to broaden access to resources and training opportunities, particularly in underserved communities (24, 25). The mandated inclusion of dual-diagnosis modules in psychology curriculum, supervised clinical rotations with ID groups, and ongoing professional development programs emphasizing diagnostic overshadowing and culturally sensitive evaluation are examples of practical ramifications.

This study had several limitations that require careful consideration. A cross-sectional design limits the ability to track changes over time or establish causal links. Furthermore, the statistical results may reflect regions with more advanced healthcare infrastructure, potentially overlooking underserved or rural regions, and introducing selection bias. Selection bias may have occurred, as psychologists with a stronger interest in intellectual disability may have been more likely to participate. Additionally, response bias cannot be excluded, as participants may have provided socially desirable answers. Future research that includes longitudinal data and community perspectives would offer a more comprehensive understanding of the barriers to accessing healthcare.

In Saudi Arabia, the use of mental health services is heavily influenced by social, cultural, and religious dynamics(such as Ruqyah (Quranic recitation) and herbal remedies). These elements can significantly influence psychologists’ attitudes, expertise, and practices in diagnosing and treating mental health conditions in individuals with intellectual disabilities. For instance, the stigma associated with mental illness, prevalent community misconceptions about mental health, and a preference for traditional healing methods may lead to delayed diagnoses or psychologists’ reluctance to propose certain interventions because of concerns about non-compliance or negative community feedback (26). Furthermore, in some traditional settings, the belief that mental illness has a spiritual basis may prompt psychologists to adjust their approach, potentially affecting therapeutic outcomes or resulting in diagnostic overshadowing.

Regional disparities in access to mental health services are particularly pronounced in rural areas where shortages often exacerbate these issues by limiting psychologists’ exposure to diverse situations and opportunities for specialized training. The necessity for culturally sensitive training and resources is thus highlighted by the fact that these cultural and social factors actively influence the professional environment and decision-making processes of psychologists working with individuals with intellectual disabilities, rather than just serving as background knowledge. Other recommendations (1) standardized national training framework; (2) integration of specialized mental health services in primary care; (3) dedicated funding for underprivileged regions; (4) formation of interdisciplinary care teams.

## 5 Conclusions

The findings of this study demonstrate the importance of clinical training and professional experience in improving the knowledge and attitudes of psychologists toward individuals with intellectual disabilities. Addressing structural barriers and fostering specialized education are essential steps for providing equitable mental health care to this vulnerable population. The results of this study highlight the value of professional experience and clinical training in enhancing psychologists’ understanding of and attitudes toward people with intellectual disabilities. It is important to emphasize that these findings represent the perceptions of psychologists rather than objective clinical outcomes. Moderate causal claims should be made given the cross-sectional nature of the data. Addressing structural barriers and fostering specialized education are essential steps for providing equitable mental health care to this vulnerable population. We emphasize the need for longitudinal and intervention studies to better understand these dynamics over time, and recommend the implementation of standardized national training programs to ensure consistent quality of care. We recommend the development and implementation of a standardized national training program in Saudi Arabia to ensure that all licensed psychologists receive adequate preparation for working with individuals with intellectual disabilities.

## Data Availability

The original contributions presented in the study are included in the article/supplementary material. Further inquiries can be directed to the corresponding author.

## References

[1] Maulik PK Mascarenhas MN Mathers CD Dua T Saxena S . Prevalence of intellectual disability: a meta-analysis of population-based studies. Res Dev Disabil. (2011) 32:419–36. doi: 10.1016/j.ridd.2010.12.018 21236634

[2] El-Hazmi MA Al-Swailem AA Al-Mosa NA Al-Jarallah AA . Prevalence of mental retardation among children in Saudi Arabia. East Mediterr Health J. (2003) 9:6–11. doi: 10.26719/2003.9.1-2.6 15562727

[3] Authority of People with Disability . Disability Report - Saudi Census 2022 (2022). General Authority for statistics. Available online at: https://apd.gov.sa/en/statistics (Accessed October 30, 2025).

[4] Alduais A Alfadda H Alarifi HS . Epidemiology of communication difficulty in Saudi Arabia: a population-based analysis using the National Disability Survey. Healthcare (Basel). (2025) 13(19):2514. doi: 10.3390/healthcare13192514 41095600 PMC12524091

[5] Almahmoud OH Abushaikha L . Prevalence and risk factors of developmental disabilities among preschool children in the Arab world: a narrative literature review. Child Health Nurs Res. (2023) 29:101–10. doi: 10.4094/chnr.2023.29.2.101 37170489 PMC10183763

[6] Bindawas SM Vennu V . The national and regional prevalence rates of disability, type, of disability and severity in Saudi Arabia-analysis of 2016 demographic survey data. Int J Environ Res Public Health. (2018) 15(3):419. doi: 10.3390/ijerph15030419 29495546 PMC5876964

[7] Alarifi S Denne L Hastings RP . Challenging behaviour and its correlates in preschool-aged children with an intellectual disability in Saudi Arabia. J Intellect Disabil Res. (2024) 68:264–76. doi: 10.1111/jir.13109 38052235

[8] Elmasry HMA Aladawy M Abd-elhamid MM . Prevalence and risk factors of intellectual disabilities in children. Egypt J Hosp Med. (2020) 81:1307–13. doi: 10.21608/ejhm.2020.112407

[9] Mazza MG Rossetti A Crespi G Clerici M . Prevalence of co-occurring psychiatric disorders in adults and adolescents with intellectual disability: a systematic review and meta-analysis. J Appl Res Intellect Disabil. (2020) 33:126–38. doi: 10.1111/jar.12654 31430018

[10] Buckles J Luckasson R Keefe E . A systematic review of the prevalence of psychiatric disorders in adults with intellectual disability, 2003–2010. J Ment Health Res Intellect Disabil. (2013) 6:181–207. doi: 10.1080/19315864.2011.65168237339054

[11] Cooper SA Smiley E Morrison J Williamson A Allan L . Mental ill-health in adults with intellectual disabilities: prevalence and associated factors. Br J Psychiatry. (2007) 190:27–35. doi: 10.1192/bjp.bp.106.022483 17197653

[12] World Health Organization . Mental Disorders (2025). Available online at: https://www.who.int/news-room/fact-sheets/detail/mental-disorders (Accessed September 30, 2025).

[13] Fan Y Fan A Yang Z Fan D . Global burden of mental disorders in 204 countries and territories, 1990-2021: results from the global burden of disease study 2021. BMC Psychiatry. (2025) 25:486. doi: 10.1186/s12888-025-06932-y 40375174 PMC12080068

[14] Mental Illness (2024). Available online at: https://www.nimh.nih.gov/health/statistics/mental-illness (Accessed October 30, 2025).

[15] National Alliance on Mental Illness . Mental Health by the Numbers. Available online at: https://www.nami.org/about-mental-illness/mental-health-by-the-numbers/ (Accessed October 30, 2025).

[16] Dell'Armo K Tassé MJ . Diagnostic overshadowing of psychological disorders in people with intellectual disability: a systematic review. Am J Intellect Dev Disabil. (2024) 129:116–34. doi: 10.1352/1944-7558-129.2.116 38411245

[17] Kildahl AN Oddli HW Helverschou SB . Bias in assessment of co-occurring mental disorder in individuals with intellectual disabilities: theoretical perspectives and implications for clinical practice. J Intellect Disabil. (2024) 28:393–414. doi: 10.1177/17446295231154119 36708367 PMC11059834

[18] Ojha V Bhatt M Singh R Goyal B . Exploring dual diagnosis: examining professional’s knowledge, attitudes, and training on intellectual disability and psychiatric disorder. Multidiscip Rev. (2023) 6. doi: 10.31893/multirev.2023ss050

[19] Emerson E Hatton C . Health Inequalities and People With Intellectual Disabilities. Cambridge University Press (2013), 1–167. doi: 10.1017/CBO9781139192484

[20] Morin D Rivard M Crocker AG Boursier CP Caron J . Public attitudes towards intellectual disability: a multidimensional perspective. J Intellect Disabil Res. (2013) 57:279–92. doi: 10.1111/jir.12008 23279318

[21] Mason J Scior K . ‘Diagnostic overshadowing’amongst clinicians working with people with intellectual disabilities in the UK. J Appl Res Intellect Disabil. (2004) 17:85–90. doi: 10.1111/j.1360-2322.2004.00184.x

[22] Reiss S Szyszko J . Diagnostic overshadowing and professional experience with mentally retarded persons. Am J Ment Defic. (1983) 87:396–402. 6829617

[23] Lunsky Y Garcin N Morin D Cobigo V Bradley E . Mental health services for individuals with intellectual disabilities in Canada: findings from a national survey. J Appl Res Intellect Disabil. (2007) 20:439–47. doi: 10.1111/j.1468-3148.2007.00384.x40046247

[24] National Academies of Sciences E Medicine . Advancing Health Care Professional Education and Training in Diagnostic Excellence: Proceedings of a Workshop—in Brief (2025). Available online at: https://nap.nationalacademies.org/read/29203/chapter/1 (Accessed August 5, 2025).

[25] Glasman LR Albarracín D . Forming attitudes that predict future behavior: a meta-analysis of the attitude-behavior relation. Psychol Bull. (2006) 132:778–822. doi: 10.1037/0033-2909.132.5.778 16910754 PMC4815429

[26] Altwaijri Y Al-Habeeb A Al-Subaie A Bruffaerts R Bilal L Hyder S . Dual burden of chronic physical conditions and mental disorders: findings from the Saudi National Mental Health Survey. Front Public Health. (2023) 11:1238326. doi: 10.3389/fpubh.2023.1238326 38089017 PMC10715453

[27] Carlisle J . Mental health law in Saudi Arabia. BJPsych Int.. (2018) 15:17–9. doi: 10.1192/bji.2017.10 PMC6020922 29953138

